# Increasing physical activity in sedentary adolescents through school-based interventions: a scoping review

**DOI:** 10.3389/fspor.2026.1736134

**Published:** 2026-06-10

**Authors:** Karin Bertills, David Johansson, Lilly Augustine

**Affiliations:** 1 Department of Behavioral Sciences and Learning, Division of Psychology, Linköping University, Linköping, Sweden; 2 Department of Social Work, School of Health and Welfare, Jönköping University, Jönköping, Sweden; 3 Department of Communication and Behavioral Sciences, School of Education and Communication, Jönköping University, Jönköping, Sweden

**Keywords:** adolescents, general population, leisure time, physical activity, school-based interventions, sedentariness, typically functioning population

## Abstract

Rising levels of physical inactivity among children and adolescents, especially among disadvantaged groups, are alarming. In response, schools should implement inclusive, evidence-based, and sustainable interventions that reduce sedentary behavior and promote daily physical activity (PA) both during school hours and in leisure time. The objective of this scoping review is to focus on adolescents with the lowest levels of PA and explore aspects that have been addressed in school-based interventions and components associated with increased PA in sedentary adolescents. Database searches were performed in PubMed, PsycInfo, and Scopus. Interventions had to be school-based, aiming to increase physical activity in participants identified as sedentary adolescents in a typically functioning population. To be included, studies had to provide information about leisure-time PA. Excluded were studies focusing solely on the general population and changes only in psychological or health-related outcomes. Rigorous evaluations of studies fulfilling all eligibility criteria were rendered in a descriptive/narrative analysis of the summarized findings of ten studies. Programs, commonly implemented during physical education (PE) lessons, often relied on PE teachers as key facilitators. Moderate activities such as walking and weight training increased the PA levels of sedentary adolescents. Theory-based motivational approaches are vital for influencing physical activity behavior. Programs that strengthen self-regulation of adolescents, such as intentions to be active and a sense of coherence, promote activity beyond school settings. Future research should establish a consistent definition of “sedentary” to enable better comparisons across studies. Sedentary adolescents should be identified as a separate group, and their perspectives and the design of the intervention should be considered in terms of intensity levels, support needs, goal setting, and progress tracking. Sedentary adolescents need engaged leaders who can provide individualized support and guidance, ideally within programs tailored to their needs. A combination of measures, self-reports, and objective measures of fitness should be used, preferably easily applicable measures.

## 1 Introduction

Programs to increase physical activity (PA) in young people in the world are urgently needed. The trend of insufficient physical activity is increasing. Global estimates show that 80% of 11–17-year-olds do not meet the guidelines for physical activity (1). Reaching a broad and diverse population, compulsory schooling provides a valuable setting for various interventions. School provides both an opportunity and a mandate to support physical activity among disadvantaged groups, i.e., children and youth without either the perceived skills, necessary support, nor identified opportunities (2). Despite numerous school-based initiatives aimed at increasing students’ physical activity levels, current interventions have proven insufficient to substantially reduce sedentary behaviors (3). The focus of this study is on interventions that engage the least physically active children and youth in a general population of typically functioning adolescents. The purpose is to investigate previous research on school-based physical activity interventions and examine how to facilitate physical activity among sedentary children and youth during school hours and leisure time.

Regular physical activity in adolescents is positively associated with improved bone health, weight status, cardiorespiratory and muscular fitness, cardiometabolic health, and cognition and reduces the risk of depression (4). According to some recommendations, children and youth aged 5–17 should engage in moderate to vigorous-intensity physical activity (MVPA) for 60 min daily, focusing on aerobic fitness, and should include muscle-strengthening activities and vigorous-intensity aerobic activities three times a week (5). The WHO guidelines are relevant for people of all abilities and valid for all irrespective of gender, cultural background, or socioeconomic status. Gerdin (6) argues that school-based physical education can be an arena for inclusion and social justice, but exclusive practices are still common. Schools can encourage physical activities, but for children and youth to reach WHO’s recommended physical activity levels, they need additional exercise in leisure time. Reduced participation in organized leisure activities is noticed in early adolescence, and interventions aiming to increase adolescent wellbeing should support physical leisure activities, with specific focus on disadvantaged groups of children and youth (7). Sedentary behaviors, defined as any waking behavior characterized by the expenditure of energy while in a sitting, reclining, or lying posture (8), increase risks for type 2 diabetes and all-cause and cardiovascular disease mortality. There is strong evidence of more pronounced negative consequences of sedentary behaviors among physically inactive people (9).

Physical activity and sedentary behaviors are established at young ages and can be tracked into adolescence and adulthood (10). Physical activity behaviors in children and youth are positively linked to mental health and self-esteem (11). Positive effects have also been found on cognitive function and academic outcomes (12). A review of interventions to promote regular physical activity and reduce sedentary behavior in a total population shows that a multifaceted approach that includes individual, community, and technology-based strategies is essential (13). Heightened sedentary behavior can be linked to both increased mental illness (e.g., depression) and lower mental wellbeing (e.g., life satisfaction and joy) (14). One report investigated school-based collaboration in mental health–promoting interventions. The identified aspects for successful implementation were solid preparation, engagement, and competence but also challenges such as disparate legislations arising from collaboration between school, health and welfare services, and social services (15). A meta-analysis of school-based interventions shows positive effects on some aspects of wellbeing but calls for improved reports on the implementation front (16). Numerous studies have addressed physical activity–enhancing and skill-building interventions, and recent reviews have reported increases in moderate-intensity physical activity (17) and fundamental movement skills (18). However, most school-based studies examining everyday physical activity behaviors in young people have neither investigated outcomes among disadvantaged groups nor examined light-intensity physical activity outcomes (19). van Sluijs et al. (20) concluded that there is a need for school-based physical activity interventions codesigned with adolescents and context-specific support to ensure effective implementation and maintenance. To evaluate health- and community-based interventions, Glasgow et al. (21) proposed the framework Reach, Efficacy/Effectiveness, Adoption, Implementation, and Maintenance (RE-AIM). The purpose of this framework is to examine sustainability, whether the intervention achieves its intended outcomes, and its potential for long-term continuation. The framework can be used to guide research about school-based interventions to increase daily physical activity. In RE-AIM, Reach encompasses the number, proportion, and representativeness of participants, and efficacy/effectiveness refers to the reported outcomes of the intervention in optimal vs. real-world settings. Adoption refers to the context and agents who initiate the intervention, implementation refers to whether the intervention was delivered as intended, and maintenance refers to whether the intervention is sustained over time (22).

Accessible physical activity is a prerequisite but does not guarantee attendance. Globally, girls are less physically active than boys, and those living with long-term health conditions or disability are often the least active (1). Aspects such as feelings of being accepted and whether the activity is adapted to make attendance and involvement worthwhile are vital to whether inactive adolescents engage in a situation (23). Additional necessary aspects related to participation refer to an individual’s motivation, sense of self, and level of skills (24). Self-efficacy plays a crucial role in shaping an individual's motivation, learning, and overall success (25). Believing in one's own capabilities to try new things and achieve goals is fundamental to developing both general and specific self-efficacy (26). The sources of self-efficacy—mastery experiences, vicarious experiences, verbal persuasion, and physiological states—are essential in building a strong sense of competence and control (27). High self-efficacy leads to greater effort, persistence, and resilience (26), while low self-efficacy can result in lower aspirations and a tendency to give up easily (28). The predictive power of self-efficacy on school performance and behavior change (26) underscores its importance as a valuable pre- and postmeasure in school interventions. One study found that students who gained most from school-based physical education (PE) were those with low grades in PE, who reported a significant increase in PE-specific self-efficacy, compared with peers who completed 3 years of school-based PE in secondary school (29).

Another important aspect to consider when promoting physical activity for the least physically active children and youth is to use inclusive approaches (2). Teachers exercise a significant impact on students’ self-efficacy by providing opportunities for success, acting as positive role models, providing supportive feedback, and devising strategies to manage stress and anxiety (30). Participation-based interventions that are tailored to individual preferences, self-beliefs, and activity competence show significant promise in enhancing physical, cognitive, and affective body functions (31), as well as social self-efficacy and engagement (32). Effective interventions require collaboration among researchers, professionals, and societal actors to support children and youth (33). The involvement of significant adults (34) and the provision of supportive environments are crucial for enabling participation (35). Schools, in particular, serve as effective arenas for promoting physical activity and should work in tandem with other social support providers to optimize participation for disadvantaged groups (36). By creating inclusive and context-specific interventions, we can better address the challenge of increasing physical activity among the most inactive groups, ultimately preventing negative health outcomes and fostering a sense of belonging and community among adolescents. Yet, it is a challenge to increase physical activity in sedentary children and youth. Aspects and experiences of disadvantaged groups of children and youth are often excluded from school-based PE research because of exclusive research methodology (37). Ethical considerations further limit the feasibility of random sampling (38), often resulting in small participant groups and findings that are difficult to generalize to broader perspectives of daily physical activity (39). Therefore, there is a need to explore previous research on effective interventions that focus on increased physical activity for physically inactive groups of children and youth.

### 1.1 Rationale

Interventions are needed to deal with the global trend of insufficient physical activity among children and youth. A challenge is to implement interventions that attract all groups of people, including those with the lowest levels of physical activity in a regular population. This study attempts to synthesize previous research on interventions conducted in school or in collaboration with schools to increase daily physical activity levels, targeting the most inactive group of adolescents.

### 1.2 Aim

The aim of this scoping review is to map research on school-based interventions targeting sedentary adolescents with the goal of increasing their leisure-time physical activity, by addressing the following questions:

1. What theories, designs, and methods have been used in school-based interventions targeting sedentary adolescents to increase their physical activity?
2. What intervention components and outcomes have been reported in school-based interventions aiming to increase physical activity among sedentary adolescents?

## 2 Methods

A scoping review was selected to get an overview of studies on the topic of how to approach sedentary adolescents to increase their physical activity during school hours and in leisure time. Scoping reviews can address research questions that extend beyond evaluations of effectiveness alone (40). A scoping review aims to map the research within a given area and identify central concepts, theories, sources and knowledge gaps where the available research does not meet the standard for a systematic review (41). Part of the aim of this study was to search for components that can improve the physical activity levels of sedentary adolescents within school hours and in leisure time. A scoping review method was decided to be the most appropriate according to the research questions. The PRISMA-ScR checklist, an extension of scoping reviews, was adhered to when synthesizing and reporting the study (42). However, no funding was received for this study and therefore this study has not been registered.

### 2.1 Search strategy

The data search was performed in August 2022 and updated in August 2024 in the following electronic databases: PubMed, PsycInfo, and Scopus. Identified key phrases were structured using Boolean operators (“OR” and “AND”), and a filter was applied (Adolescent). Reference lists of included studies were manually searched, aiming at identifying relevant studies that were not included in the electronic searches (see Supplementary Material 1).

### 2.2 Eligibility criteria

The eligibility criteria were developed from the research questions in reference to the population, intervention, comparison and outcome (PICO) protocol (43). Guided by the aim and research questions to review school-based interventions targeting sedentary adolescents, PICO was considered the best fit.

#### 2.2.1 Population

Participants had to be explicitly identified as being sedentary but otherwise belonging to a typically functioning population of adolescents. Studies with participants having any reported characteristic that might hinder them from being physically active (e.g., being obese or having a disability) were excluded. The operationalization of sedentary adolescents corresponds in part to Schneider, Dunton, and Cooper (44) criteria: (1) not being part of a sports club or sports team and (2) not having more than three 20-min bouts per week of vigorous physical activity and more than five 30-min bouts per week of moderate physical activity. Studies were included if participants were between 13 and 18 years of age and enrolled in school. Studies with a wider age range needed to have an identified subgroup with participants 12–18 years of age and to provide stand-alone data for this subgroup to be included.

#### 2.2.2 Intervention

To be included, the intervention had to be school based and performed within a school setting or being monitored by the school. The intervention had to aim at increasing daily physical activity levels during school hours and/or in leisure time. Eligible interventions ranged from trying to teach the importance of physical activity, influencing psychological concepts associated with physical activity, and performing physical activity to start monitoring such activity by, for example, the use of a pedometer. Interventions using multiple aspects within the described range were eligible.

#### 2.2.3 Comparison

Few studies explicitly targeted sedentary adolescents as a predefined category; instead, eligibility was determined by how individual studies operationalized and described participants’ sedentary behavior. Included studies rarely used formal experimental design and therefore no clearly defined comparison groups. Interventions ranged from teacher training and student education to increased opportunities for PE or specific PE classes. This heterogeneity in methods is relevant to the aim and is feasible for a scoping review.

#### 2.2.4 Outcome

Included studies needed to provide results about the adolescent's leisure-time physical activity with at least one pre- and postmeasurement. The leisure-time physical activity could be measured both by objective measurements such as pedometers and with self-reports. Continual measures as well as cross-sectional measures of physical activity were accepted. Changes in psychological concepts or health-related parameters such as blood pressure were excluded.

### 2.3 Study selection

**Figure 1:**
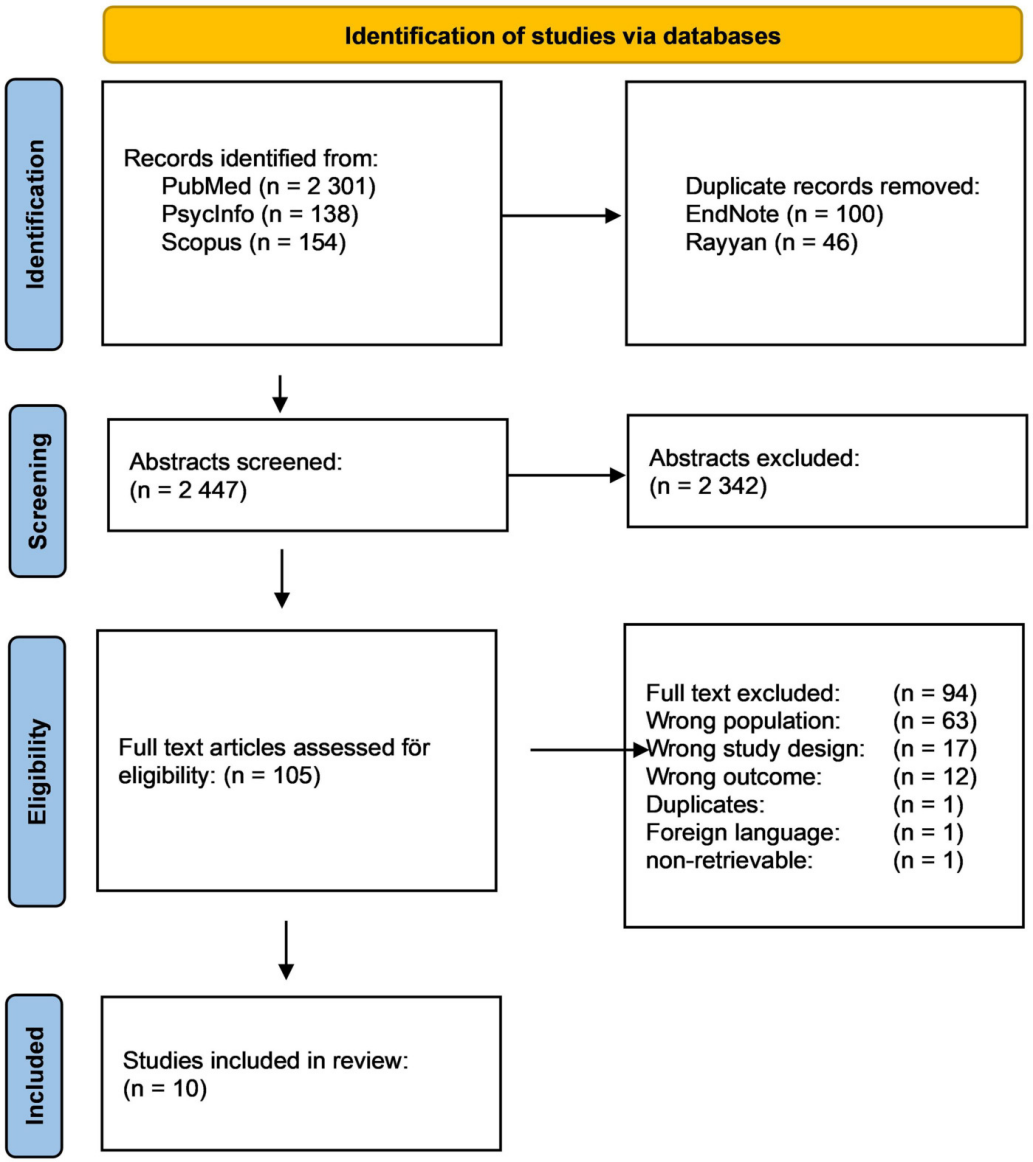
Flow diagram for the study selection process.

The data searches (see Figure 1) were exported to EndNote, performing an initial deletion of duplicates and then exported to Rayyan (45) to identify additional duplicates. Three researchers conducted screening, one screened all abstracts (DJ), the other two, half each (LA and KB). LA screened most full texts, while KB and DJ each screened roughly half each. A third rater resolved inclusion disputes.

### 2.4 Data extraction

Extraction from included studies (*n* = 10) was guided by the original guidelines and the enhancements described in (46) to identify relevant studies and research question, chart the data, summarize findings, and finally report the results, using Excel. Prior to extraction, the protocol was pilot-tested and revised to increase usability and relevance for the research questions (see Supplementary Material 2). The final version included the following: study identification, country, aim, research question, target group, recruitment, sampling strategy, participant characteristics, definition of the sedentary group, support provision, design, follow-up, measurements (leisure activity, intervention, physical activity, social/mental aspects), method, theory, type of intervention, outcomes, conclusions, and practical implications. Two researchers independently extracted data from half of the studies each, and half (48%) of these studies were dually conducted ensuring quality in the process.

### 2.5 Data analysis

To understand what theories, designs, and methods were used in school-based interventions aimed at increasing physical activity during school hours and/or in leisure time, a narrative analysis was conducted summarizing the findings of the 10 included studies. This involved describing and interpreting reported intervention outcomes without statistical pooling or formal evidence appraisal. To support the narrative analysis, reported effect sizes were grouped descriptively into categories of large, medium, small, or no change (see Supplementary Material 3). However, as many included studies did not report standardized effect sizes and used different measurement approaches between pre- and postassessments, effect estimates were derived from reported results, when possible, to facilitate descriptive comparison across studies.

## 3 Results

The findings from this scoping review of 10 studies will summarize research about school-based interventions to increase physical activity levels among sedentary adolescents. To simplify the references, each study was given an identification number (ID#).

### 3.1 Characteristics of selected studies

Included articles were published between 2004 and 2024, and all were quantitative in design, with one using a mixed design and one using register data. An overview of the included studies is provided in Table 1.

**Table 1:** Description of included studies.

ID#	Title	Country	Nr of part.	Age	Population	Sample	Theory	Nr of sch.	Int. length	Follow-up
#1	A physical activity intervention for Brazilian students from low human development index areas: A cluster-randomized controlled trial	Brazil	1,085 *I* = 548 *C* = 537	13–14	Low SES	Strat.	Yes	6	16 weeks	Pre/post
#2	Evaluation of a Computer-Tailored Physical Activity Intervention in Adolescents in Six European Countries The Activ-O-Meter in the HELENA Intervention Study	Belgium	1,050 *I* = 581 *C* = 469	15	Reg.	Strat.	Yes	49	12 weeks	Pre Post1, 4 weeks Post2, 12 weeks
#3	Factors predicting behavioral response to a physical activity intervention among adolescent females	USA	63	15	Girls	Strat.	Yes	2	40 weeks	Pre/post
#4	A controlled evaluation of a school-based intervention to promote physical activity among sedentary adolescent females: project FAB	USA	47 *I* = 25 *C* = 22	15	Girls	Conv.	Yes	1	16 weeks	Pre/post
#5	Salutogenesis as a framework for improving health resources of adolescent boys	Poland	199 *I* = 84 *C* = 115	13	Boys	—	Yes	1	60 weeks	Pre/post
#6	Effects of Epstein's TARGET on adolescents’ intentions to be physically active and leisure-time physical activity	Spain	447 *I* = 223 *C* = 224	14	Reg.	Conv.	Yes	8	12 weeks	Pre Post1, 12 weeks Post2, 24 weeks
#7	Impact of the "Planning to be Active" Leisure-time Physical Exercise Program on Rural High School Students	USA	240 *I* = 143 *C* = 97	15	Rural	Strat.	Yes	2	8 weeks	Pre/post
#8	A pragmatic multisetting lifestyle intervention to improve leisure-time physical activity from adolescence to young adulthood: the vital role of sex and intervention onset time	Iran	1,251 *I* = 319 *C* = 932	12–18	Reg.	Random	No	12	16 years	Pre 5 post, 3-year intervals
#9	Impact of a school-based physical activity intervention on fitness and bone in adolescent females	USA	122 *I* = 63 *C* = 59	15	Girls	Conv.	No	2	40 weeks	Pre 5 post, 20-week intervals
#10	The association between childhood motor performance and developmental trajectories of sport participation over 5 years in Danish students aged 6–16 years old.	Denmark	1,547 *I* = 882 *C* = 665	11–18	Reg.	Conv.	No	6	5 years	Pre Post, weekly for 4 years and 3 months

Nr of part., number of participants; Nr of sch., number of schools; Int. length, intervention length; I, intervention group; C, control group; Reg., regular; Conv., convenience; Strat., strategic.

### 3.2 Theories

Examining which theories were applied in school-based interventions aimed at increasing daily PA among sedentary adolescents, One use of theories was in the selection of instruments. Validated scales were used to investigate PA-related aspects as potential mediators of PA (47–49). Another use of theories was in the construction of teaching strategies (50–52). Psychosocial determinants of PA were theory-based in feedback that was tailored to fit individual students (53). No theory was pronounced in three studies (54–56) (see Table 1).

Theories and models represented in the articles with theory-based measurements were Socioecological theory and the concept of the Health Promoting Schools (47), Social Cognitive Theory (SCT) (48, 49, 52, 53), the theory of planned behavior, and the Attitude, Social Influence, and Self-Efficacy model (53). A salutogenic approach was combined with Hellison's Teaching Responsibility through Physical Activity model in an intervention delivered by PE teachers (50). Epstein's TARGET framework guided the development of an intervention to promote motivational PE climates of mastery (51), and an exercise program was based on SCT to promote leisure-time PA (52).

### 3.3 Designs

In reviewing the study designs, it was found that one study specifically addressed sedentary adolescents as one group (3), while the other studies compared intervention with control groups. Table 1 shows that four studies were large-scale comparisons with more than 1,000 participants (47, 53, 54, 56), one had a total number of 447 participants (51), and five had participants ranging from 47 to 240 (48–50, 52, 55). Countries represented in the studies were Belgium (53), Brazil (47), Denmark (56), Iran (54), Poland (50), Spain (51), and the United States (48, 49, 52, 55). Three studies focused exclusively on adolescent girls (48, 49, 55), one focused on adolescent boys (50), one on students attending a rural high school students (52), one on students from areas with low human development index (47), and four targeted regular high school student populations (51, 53, 54, 56). Participants were recruited in schools in all studies, but in one, recruitment was made through health services (54). Four studies used convenience sampling (49, 51, 55, 56), four strategic sampling (47, 48, 52, 53), one random sampling (54), and one did not mention the type of sampling (50). All studies but one (48) used an experimental design comparing intervention with control groups. The analyses were compared between clusters (47, 56), genders (51, 54), and between countries (48).

### 3.4 Methods used for the intervention outcome

Focusing on increased PA in leisure-time findings shows that self-reports were used in all studies measuring numerous aspects. Physical and psychosocial factors related to motivation for exercise and efforts made. Physical factors of time, frequency, and intensity of PA during school hours and in leisure time were scored or recalled in diaries/logs. Students reported frequency of MVPA (50), leisure-time PA (51), type and intensity of PA (52), frequency and intensity of weekly PA (54), and estimated their average daily minutes of MVPA (55). Parents regularly reported the amount and type of organized leisure-time activity in which their child had participated during the previous week (56).

As inclusion criteria required both pre- and postintervention measurements, all included studies employed designs that allowed for such assessments. The main differences between them concerned the duration of the intervention. Five studies used pre- and postmeasures with follow-up after 2 (52), 4 (47, 49), 10 (48), and 15 months (50) (see Table 1). Two 12-week interventions had baseline data with two follow-ups: one after 1 and 3 months (53) and the other after 3 and 6 months (51). Three studies used repeated measures over an extended period of time. The first study used register data in 3-year intervals over 15.9 years (54). The second one collected data over a 3-year period at baseline with follow-ups after each school semester (55), and the third collected data weekly for a total of 4 years and 3 months (56).

Table 2 shows the types of intervention activity and outcomes. Subjective self-reports of physical outcomes (time, frequency, and type of PA) were recorded in all studies and objective physical outcomes in three studies (49, 50, 55). Psychosocial outcomes were measured in terms of PA-related preferences (47), theory-based feedback (53), benefits, competence and support (48, 49), sense of coherence (50), and intentions (51).

**Table 2:** Intervention type and outcome measurement.

ID#	Type of activity	Support provision	Type of intervention	Intervention outcome measured—physical	Intervention outcome measured—Psychosocial
1	Opportunities for PA in school	Staff member	Combination of teacher training, PE teacher specific training, opportunities for PA in school, and health education	Time (min/week) and number of types of PA (active commuting), PA in leisure time	Preference for leisure-time PA and stages of PA behavior change
2	Tailored PA advice based on profiling	Computer tailored	Self-reports into the diagnostic tool of psychosocial aspects related to PA followed by feedback with PA guidelines	Time (min/week) of each moderate and vigorous activity	Tailored feedback: attitudes, self-efficacy, social support, knowledge, perceived benefits, and barriers related to PA
3	PA in a PE setting and classroom health education	PE teacher and research staff (health)	4 days of PA (40 min) and 1 day of health education	Duration (min) and type of PA in vigorous vs. moderate PA	Perceived benefits from PA, self-efficacy for PA, social support for PA, perceived barriers for PA, and PA enjoyment
4	PA in a PE setting and strategies for change; self-monitoring, goal setting, and problem-solving	PE teacher and aerobic dance instructor	4 days of PA (40 min) and 1 day of lecture/discussion PA-enhancing strategies	Cycle ergometer (VO_2peak_ L/min), 2-day PA recall (30-min bouts into METs), and daily life PA	Self-efficacy, barriers, social support, and enjoyment
5	PE teaching strategy and student planning leisure-time PA	PE teacher	4 PE lessons/week teachers used a strategy to improve student self-control/-responsibility	20-m shuttle-run test. Frequency of leisure-time MVPA in relation to self-assessments of fitness	Sense of coherence; manageability, meaningfulness, and comprehensibility
6	PE teaching strategy inviting students to influence their own learning	PE teacher	Strategies during PE class to promote intention to be physically active and leisure-time physical activity	Group and gender (independent) and leisure-time physical activity levels (dependent)	Intention to be physically active
7	PE lesson	Investigator	Behavioral skill-building, based on theory, and 1 PE lesson/week	Number of days per week of 30 min blocks of MVPA	—
8	Nine 45-min classes	Volunteer students and parents	Strategies to promote a healthy lifestyle	Leisure-time PA	—
9	PA based on student input in a PE setting and health education	Supervisor	4 days of PA (40 min), 1 day of health education, and digital self-monitoring	Cycle ergometer, 3-day PA recall (30-min bouts into METs), and heart rate monitor	—
10	PE lesson		270 min extra PE/week in ages 5–12	Frequency and type of sport participation	—

PA, physical activity; PE, physical education; MET, metabolic equivalent, energy expenditure of an activity, MVPA, moderate to vigorous activity.

**Figure 2:**
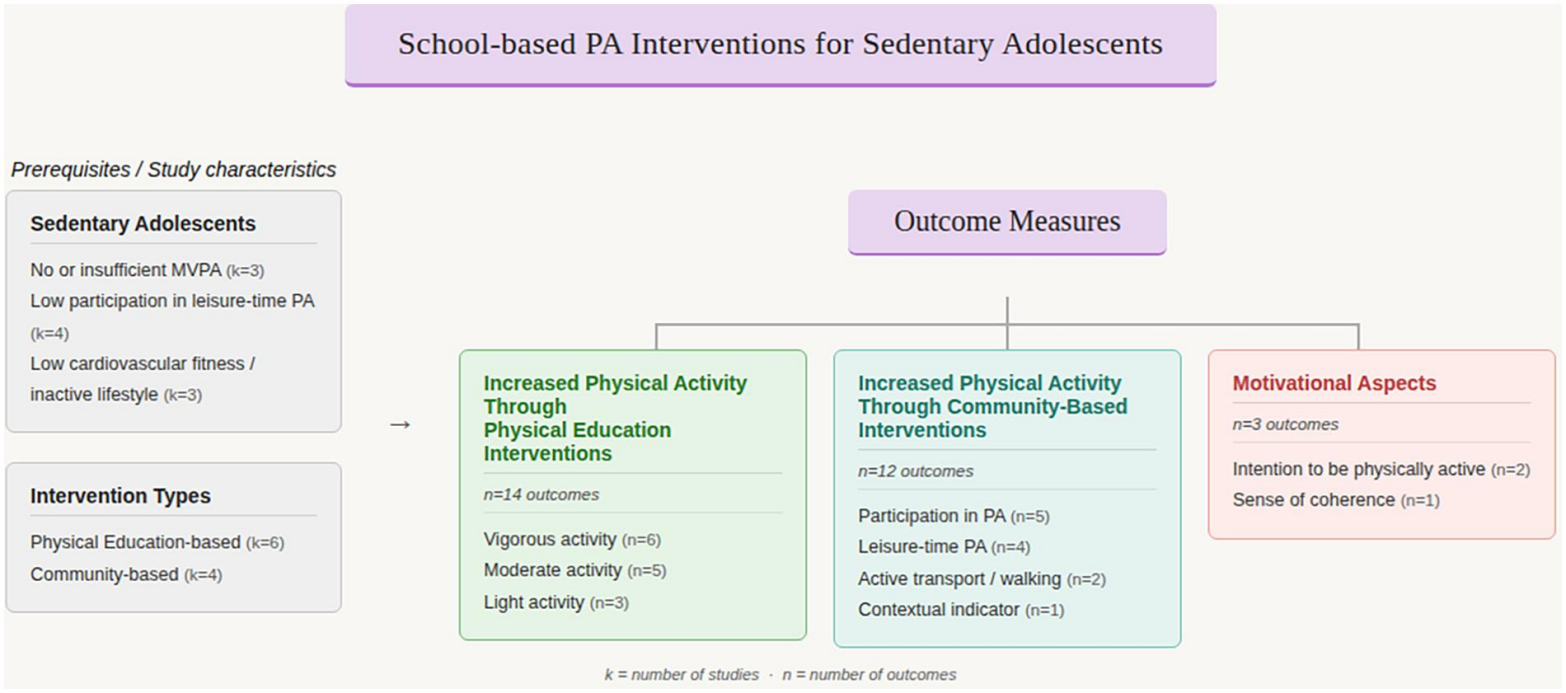
School-based PA interventions for sedentary adolescents.

School-based interventions aiming at increasing PA among adolescents present a wide range of reported outcomes with various ways to display the variables measured and statistical analyses used. The inclusion criterion for this scoping review required that studies reported outcomes for sedentary groups of adolescents separately from the total sample. The review showed that findings relating specifically to sedentary groups of adolescents were sometimes embedded in variables that needed to be scrutinized for further interpretation, for example, the finding that the control group had higher drop-out rates. Figure 2 shows a visual map of identified groups of sedentary adolescents, intervention types, and outcome domains reported in the included studies categorized into physical education-based, community-based, and motivational aspects.

### 3.5 Intervention outcomes influencing sedentary adolescents’ physical activity levels

Outcomes (*n* = 29) were first identified in the different studies and categorized according to outcome type (see Supplementary Material 3), for detailed outcome descriptions and reported changes as reported or estimates derived from reports in original studies. Two types of interventions were identified: interventions in school-based physical education settings (*k* = 6) and interventions in community settings in collaboration with schools (*k* = 4). Across the 29 identified outcomes, most studies reported changes in PA measures, primarily concerning physical outcomes focusing on vigorous (*n* = 6), moderate (*n* = 5), light (*n* = 2), and other activity (*n* = 7). Motivational outcomes were reported in two studies (50, 51) measuring intention to be active (*n* = 2) and sense of coherence (*n* = 1). Both studies were conducted within school-based physical education settings (see Figure 2).

### 3.6 Increased physical activity through physical education interventions

PE lessons were a common setting to implement programs aimed at sedentary adolescents (*k* = 6) (see Table 2) with a total of 14 pre/post measurements (see Figure 2), 6 vigorous, 5 moderate, and 3 light activities. Vigorous activity was commonly used as an outcome measure to assess changes in physical capability in school-based interventions targeting sedentary adolescents. Precardiorespiratory fitness and postcardiorespiratory fitness were measured using cycle ergometers (49, 55) and a self-reported diary of previous 1–7 day PA recalls (49, 52, 55). In addition, cardiorespiratory fitness was assessed from 20-m shuttle-run tests (20SRTs) and a recall of previous month frequency of MVPA (50). Several studies reported changes in moderate and vigorous activity in the intervention groups. Sedentary students engaged in moderate exercise, whereas in the control group, the number of sedentary students increased (52). Teaching strategies with a salutogenic approach in the setting of PE lessons for boys was used to increase out-of-school PA (50).

Changes in moderate activities, for example, weight training in leisure time among sedentary adolescents, were reported in four studies (48, 49, 52, 55). Moderate-intensity activities in leisure time were more accessible to adolescent girls who initially reported less environmental barriers, and girls who reported internal barriers to PA reported increased moderate PA (48). Average daily minutes in moderate activity remained stable among girls in the intervention group, whereas it declined in the comparison group (55). Reports of girls on the number of lifestyle activities such as taking the stairs instead of the elevator were higher in the intervention group than in the control group, which reported little change (49). Findings from an intervention designed for girls included health education and PE lessons 5 days/week. The study reported higher levels of leisure-time vigorous activity. Participants initially exercising for 11 min reported levels totaling 26 min of leisure-time vigorous PA after 20 weeks (48).

The PE teacher played a central role in delivering the programs. Applying health education was associated with higher vigorous activity in girls (49). Higher activity levels remained for the intervention group in the same type of setting after two semesters (55). PE teachers were trained to apply strategies for locus of control, which was more profitable for boys than for girls, but intention to be physically active was higher, and leisure-time physical activities were reported to be higher in the intervention group than in the control group (51).

### 3.7 Increased physical activity through community-based interventions

The additional studies (*k* = 4) incorporated a community-based intervention in collaboration with schools to increase PA. Eight of the 12 outcomes focused on PA behaviors, with five demonstrating positive changes in PA. Changes in activity-engagement resulting from PA participation were focused in four of the 12 outcomes. For example, one additional PE lesson per week was implemented and followed up after 5 years. The results reported an association between high total motor performance and future leisure-time sports participation for the two most active sports trajectory groups compared with the group with the lowest motor performance at baseline. School type mattered when comparing the outcomes of extended time for PE lessons between regular high school students and students from sports schools (56).

Feedback was considered a vital component. One computer-based intervention with screening and subsequent personalized feedback investigated changes in PA behaviors. Computer-tailored advice as compared to generic advice was reported to encourage a subgroup of adolescents who were identified as inactive (not meeting the 60 min/day MVPA guidelines) to increase their moderate activity such as walking and vigorous activity in leisure time as well as their total MVPA. Personalized feedback for the inactive group resulted in increased cycling for transportation after 1 month but not after 3 months (53). Giving feedback through gamification, a PA-promoting program offering opportunities for PA in school resulted in increased weekly time in popular games, and total time and number of weekly MVPAs in the intervention group (47) in relation to the control group. A third study, utilizing a multisetting intervention incorporated face-to-face counseling, written materials, public health education, and the promotion of health policies to facilitate healthy lifestyles among the intervention group, as part of strategies implemented at the family, school, and community levels. The results showed increased leisure-time PA levels in younger boys following early intervention onset, but not in other groups (54).

### 3.8 Motivational aspects in interventions

Although only two studies incorporated pre- and postmeasures concerning motivational aspects (50, 51), most studies developed instruments and methods based on motivational theories/models targeting potential psychosocial mediators. Students’ self-reported data were used to assess psychosocial factors related to PA during school hours and/or in leisure time. As part of applying self-regulation skills, students in a rural school reported self-set goals. A total of 83% of the students set goals that focused on moderate-intensity PA, for example, walking, cycling, and weight training (52). The most investigated psychosocial aspects (see Figure 2) associated with PA in the studies were self-efficacy, social support, perceived barriers (48, 49, 53), perceived benefits (48, 53), and PA enjoyment (48, 49). Other PA-related psychosocial aspects reported were participation in popular games, weekly time in PA, and number of PAs per week (47), attitudes to PA and knowledge about PA (53), sense of coherence, manageability, meaningfulness, and comprehensibility (50), and intention to be physically active (51).

Motivational aspects formed the basis for a majority of the interventions (7 of 10) in the sense that instruments, teaching strategies, and feedback were theory based (see Table 1). Outcomes of psychosocial factors, however, were reported as modest by the original authors. The study reported no changes in the psychosocial aspects measured (49), and self-reports were reported to be more valid for older adolescents than for younger ones (53). Girls’ compliance with weekly web-based logs over time was seen to be declining (48). The retention rate was 47% in the 3-month intervention that compared computer-based individual feedback with controls who received generic advice (53).

## 4 Discussion

School-based interventions to increase physical activity rarely target or report results from disadvantaged groups because of an exclusive research methodology (37). In this scoping review, sedentary adolescents’ PA levels were reported to change across most of the identified interventions. The included studies suggested that school-based PA interventions targeting sedentary adolescents as a separate group reported more favorable outcomes than interventions in the general population, which commonly show modest results. Interventions including active adolescents may have a limited scope to demonstrate changes in PA levels, meaning that PA levels may not differ from baseline to follow-up. Sedentary adolescents are hard to reach, but the included studies report changes in PA levels following school-based interventions and suggest that efforts to involve sedentary adolescents in leisure-time PA are worth the resources, time, energy, and financial support. Here follows a presentation of aspects that have been addressed in research and a discussion about vital components to enhance PA in sedentary adolescents.

MVPA is an established way to measure the frequency and intensity of physical activity. One criterion for this study was that participants were explicitly identified as sedentary. The definitions of sedentary adolescents displayed in Table 3 indicate a large spectrum of aspects that were either assessed physically and/or self-reported. Sedentary, inactive, unfit, and “not a regular exerciser” were similar terms used, and several terms expressing insufficient levels of MVPA were also used, for example, “some or no numbers of 30-min blocks of participation in hard activity.” Consensus on how to best define sedentary cannot be drawn from the investigated studies. Based on the results of this study, the main message is that groups of sedentary adolescents should be identified and studied as a separate group with results clearly displayed to justify the level of success and explain what works for what groups of adolescents.

**Table 3:** Included studies, definitions of sedentary adolescents.

ID#	Author	Definition of sedentary adolescents
#1	Barbosa Filho et al. (47)	Inactive students (0 min/week of MVPA)
#2	De Bourdeaudhuij et al. (53)	Inactive—not complying with the recommended activity levels of at least 60 min of MVPA per day
#3	Dunton et al. (48)	No participation in sports team, not a regular exerciser, insufficient levels of recommended activity of MVPA, and relatively low cardiovascular fitness (VO_2peak_ at or below age-specific 75th percentile) but with ability to exercise without restrictions
#4	Jamner Schneider et al. (49)	Physical fitness cardiovascular fitness peak oxygen uptake (VO_2peak_, L/min) and some or no numbers of 30-min blocks of participation in hard activity
#5	Bronikowski and Bronikowski (50)	“Unfit”—participating in MVPA less than once a week
#6	Cecchini et al. (51)	Participating in leisure-time physical activity for 20–30 min during free time over the past 3 months 1–2 times per week
#7	Hortz and Petosa (52)	Engaging in 0 days of MVPA
#8	Parvin et al. (54)	No regular, strenuous, and self-rated physical activity in the past week
#9	Schneider et al. (55)	Insufficiently active in recommended MVPA and relatively low cardiovascular fitness (VO_2peak_ at or below age-specific 75th percentile) but with ability to exercise without restrictions
#10	Lykkegaard et al. (56)	Average weekly sports participation of less than 0.40 sessions

Recommended activity of MVPA = three 20-min bouts per week of vigorous physical activity and five 30-min bouts per week of moderate physical activity; VO_2peak_ = highest amount of oxygen consumed at peak exercise. Quality: H, High; M, Medium, no low-quality studies included.

The result that vigorous PA appears to be an effective component in measuring increased physical activity during school hours and in leisure time among sedentary adolescents is not surprising. Participants may start at zero at baseline, and if they do any vigorous activity at follow-up, there is an obvious improvement. Some sedentary adolescents may prefer short, intense workout, but high-intensity activities may also require additional effort, like changing clothes, and may trigger feelings of aversion because of painful or embarrassing bodily reactions like sweating. One may ironically argue that any physical activity is better than none at all when studies report an increase of 15 min of weekly leisure-time vigorous activity after a whole school term of daily PA intervention. However, activities at moderate levels seem to be more attractive or relevant for sedentary adolescents. Favorable or stable patterns in the intervention groups as compared to controls were expressed in terms of the control group showing declining or no change in MVPA, or a growing number of inactive students. In this scoping review, studies reported increased PA in interventions specifically targeting sedentary girls, for example, lifestyle activities like walking for transportation. A majority of students in a rural school set goals focusing on moderate intensity, for example, weight training. Inactive students in a computer-tailored PA intervention reported increased walking in leisure time. Organized leisure-time activities should support disadvantaged groups of adolescents (7). Structured activities may be accessible but not adapted to make attendance and engagement worthwhile (23). The findings in this review highlight that everyday PA behavior is not just about access or opportunity, it is also about mental readiness and awareness, and additionally about habitual formation and a sense of competence or capacity.

Heavy reliance on self-reports covering a wide range of physical and psychosocial variables was found. One study noted that self-reports were more valid among older adolescents (53). Children often overestimate their abilities, and with age, they become more capable of recognizing their limits, monitoring their performance, and responding to feedback (57). Seen from the perspective of self-reporting PA, objective measures are needed to confirm validity. Self-reports are prone to biases, for example, social desirability bias and recall bias (58), when estimating previous frequency and intensity of PA in logs/diaries. In a school context, both objective and subjective measures should be easy to apply. One combination found was an objective measure of cardiorespiratory fitness from 20-m shuttle-run tests and recalls of previous frequency of MVPA (50). Recalls in logs/diaries seem to be necessary to report PA, which raises questions about intervention time frame. Considering the target population in this review, which is sedentary adolescents, it can be argued that a longer time frame would be needed to inculcate regular habits of increased PA in this population. However, over time, a declining compliance with self-reports was reported in a 40-week period (48). A shorter time span of 12 weeks with two follow-ups reported increases in PA in two of the screened articles (51, 53), but an ideal time frame for school-based PA interventions targeting sedentary adolescents remains unrevealed.

Psychosocial measurements, teaching strategies, and feedback were found to be theory based. Criteria for inclusion in this review related to sedentary adolescents in a typically functioning population, without reported PA-restricting characteristics such as obesity or disability. This population may have the required knowledge, skills, and opportunities to follow recommendations given for PA, but reasons for inactivity are numerous. The theories, frameworks, and models used in screened studies show that motivation (59) is vital for promoting everyday PA behavior. However, the multitude of psychosocial aspects measured makes outcomes difficult to interpret. Motivational aspects were considered vital in the design of PA interventions, but as outcome measures, only two reported changes: intention to be active (51) and sense of coherence (50). Determinants of whether adolescents with insufficient levels of PA will change their behaviors to a more active lifestyle relate to a variety of individual and environmental factors. Considering the benefits of increased daily PA for sedentary adolescents, it can be argued that modest outcomes of psychosocial aspects may still be considered meaningful. To show plausible changes in everyday PA behaviors, we suggest that the number of psychosocial aspects measured should be minimized and adapted to specific PA contexts.

PE teachers can play a significant role in influencing students’ intentions to be physically active and their physical activity levels (51). School-based PE can be inclusive and mastery-oriented, but PE settings can also be competitive and performance-oriented (6). In one study, it was found that teachers attended 30 h of theoretical and practical training before implementation in school, and also organized weekly seminars during the 12-week implementation period to share their experiences and discuss videotaped lessons. In creating a mastery motivational climate (as opposed to competitive), teachers recognized, evaluated, and rewarded individual progress in private and independently of student skills or pace. Students reported that they maintained positive outcomes 3 months after the intervention. This example illustrates that PE teachers may impact increased leisure-time PA by providing an inclusive teaching approach that offers opportunities for success (30) and by being a significant and supportive adult (34). Other interventions delivered by PE teachers implemented programs that promoted self-regulation, fitness knowledge, self-efficacy, and outcome expectancy value. School-based interventions specifically designed for sedentary adolescents were found in school systems with PE lessons each school day. This is not applicable in other school systems, yet the studies included in this review suggest that the regular structure of PE classes can be leveraged more effectively to reach sedentary students. Mandatory education, reaching all students, makes schools a common setting for implementing PA-promoting interventions (60). However, irrespective of the school system, PE lessons need to be complemented with leisure-time PA in order for children and youth to reach the recommended PA levels of 60 min of MVPA per day and additional vigorous physical activity three days per week (5). The included studies suggest that PE lessons are a key setting for PA interventions, and that PE teachers are key agents to implement teaching strategies that encourage students to influence and control their own PA during school hours and in leisure time. These findings are in agreement with the conclusion of van Sluijs et al. (20) that school-based PA interventions need context-specific support and should be codesigned with adolescents.

## 5 Strengths, limitations, and future research

The primary strength of this scoping review was that sedentary adolescents in a typically functioning population were targeted. Interfering with inactivity at young age is of high priority since future negative effects of sedentary behaviors are more pronounced in this population (9). The limitations of this study can be found in the choice of the review, which is a scoping review; the data searches resulted in an overinclusion of PA interventions. Two-thirds of full texts were excluded because of a wrong population group. Although the selection of screened studies was limited, a scoping review enables the inclusion of a broader range of evidence. Had a systematic review been required, conducting this study might not have been feasible. Full-text screening needed thorough scrutinization and strict conformity to eligibility criteria to detect a sedentary group. To avoid selection bias, all full texts were screened by two reviewers and conflicts resolved by a third. To identify intervention components and due to a large number of outcomes measured, an overview was needed. Effect sizes as reported in the studies or as estimated based on results reported by the original authors are presented in Supplementary Material 3 to provide additional detail on the reported outcomes. Given the limited number of studies found, the results of this study should be interpreted cautiously. Notably, a majority of the full-text articles assessed for eligibility were excluded because of missing identification or data about the target group. This indicates that a definition of sedentary adolescents should be established because they need to be identified and investigated as a separate group.

The fact that three studies from the same intervention were included in this review may be seen as a limitation. This intervention included only girls and was delivered as daily PA in particular PE lessons. However, girls are globally less physically active than boys (1). Targeting only girls highlights the importance of tailoring interventions according to individual preferences, self-beliefs, and activity competence (31) to encourage presence and engagement. Other groups of sedentary adolescents were identified in the general population studied, for example, students in schools with low socioeconomic status, students in rural schools, or only male students. Further research is needed to develop theory-based teaching strategies that promote motivational PE climates of mastery and render individually tailored advice to support positive PA behaviors (59, 61).

## 6 Conclusion

This scoping review aimed to map research about school-based interventions, and the findings in a majority of the included studies indicated that sedentary adolescents increased their physical activity during school hours and in leisure time. The group of sedentary adolescents was identified in many different ways by either self-reports and/or by assessing physical capacity. Measuring pre/postoutcomes of vigorous physical activity showed improvements. However, physical activity at a moderate- or light-intensity level seemed more relevant when interventions were designed for sedentary students. Motivational aspects were considered vital for influencing physical activity behaviors and were used in the planning of interventions for the construction of instruments, teaching strategies, and feedback. Theory-based training sessions before implementation may provide the implementors with practical tools and strategies to encourage adolescents and provide motivation to them to increase their physical activity levels. Opportunities to share their experiences and challenges with students during the period of implementation may improve the operational skills of the implementors, such as classroom management and how to best provide support for sedentary adolescents. Implementing school programs to promote self-regulating skills, for example, intentions to be physically active and sense of coherence, can inspire adolescents to be physically active in leisure time and thereby reach recommended levels of moderate to vigorous activity. Potentially mediating psychosocial aspects were found to be numerous in this review, but most appeared inadequate as measures of intervention outcomes. The reviewed studies showed a heavy reliance on self-reports.

Engaging sedentary adolescents in school-based interventions to increase daily PA during school hours and in leisure time requires efforts that go beyond what is known from previous research. The findings in this review suggest that sedentary adolescents should be identified and studied as a separate group. Preferably, they should be listened to before the period of implementation in terms of intervention time frame, type of activities, frequency, preferences for activities at moderate vs. vigorous intensity, support needs, and how to set goals and document progress. Regular support, i.e., feedback and feed-forward to reach individual goals, can be provided through PE teaching strategies or digitally tailored feedback. Sedentary adolescents may need knowledge and experience to understand bodily responses like sweating from physical activity. In addition, they may need tools to find leisure-time activities that may help them reach the recommended levels of daily physical activity. Elements of the type of activity and the frequency and intensity of activities that they perceive as meaningful in leisure time would complement outcomes of school-based physical activity interventions.

The findings in this scoping review suggest that future research should formulate a uniform definition of “sedentary.” Initially involving sedentary adolescents in the design can provide valuable input before implementation. Engaging leadership is required, if feasible in specific physical activity programs for sedentary adolescents. Subjective self-reports should be simple to use and combined with easily applicable objective measures of physical fitness, for example, a 20-m shuttle-run test. Changes in physical activity behaviors should be measured more frequently than only before or after the intervention. Relying on only two measurement points captures only linear trends (e.g., from good to better). Incorporating multiple measurement occasions would enable the examination of non-linear trajectories across different groups of adolescents, thereby allowing a deeper understanding of what works, for whom, and under which conditions. Qualitative feedback from participants at follow-up would further inform research about what triggers sedentary adolescents to start being physically active and what makes them sustain healthier physical activity behaviors.
