# Supplementary material for: Increasing physical activity in sedentary adolescents through school-based interventions: a scoping review
Source: Front Sports Act Living. 2026 Jun 10;8:1736134. doi: 10.3389/fspor.2026.1736134 (PMC13291142; doi:10.3389/fspor.2026.1736134)
Supplement: Supplementary file 1 [file Supplementaryfile1.docx]

# Supplementary material 1

**Search strategy in PubMed, PsycInfo and Scopus**

| ***PubMed*** | **Key phrases** | ***Number of hits*** |
| --- | --- | --- |
| #1 | ti/ab (Physical activity) OR sedentar* | 663865 |
| #2 | ti/ab intervention OR promotion | 10478716 |
| #3 | ti/ab school-based OR schoolbased OR (school based) | 1229473 |
| #4 | ti/ab leisure-time OR free-time OR extracurricular | 279 807 |
| #5 | #1 AND #2 AND #3 AND 4 | 8531 |
| #6 | Add filter: Adolescent (13-18 yrs) | 2054 |

| ***PsychInfo*** | **Key phrases** | ***Number of hits*** |  |
| --- | --- | --- | --- |
| #1 | ti/ab (Physical activity) OR sedentar* | 118345 | |
| #2 | ti/ab intervention OR promotion | 590785 | |
| #3 | ti/ab school-based OR schoolbased OR (school based) | 333171 | |
| #4 | ti/ab leisure-time OR free-time OR extracurricular | 15307 | |
| #5 | #1 AND #2 AND #3 AND 4 | 416 | |
| #6 | Add filter: Adolescent (13-17 yrs) | 128 | |

| ***Scopus*** | **Key phrases** | | ***Number of hits*** |
| --- | --- | --- | --- |
| #1 | ti/ab (Physical activity) OR sedentar* | 562958 | |
| #2 | ti/ab intervention OR promotion | 1962357 | |
| #3 | ti/ab school-based OR schoolbased OR (school based) | 264278 | |
| #4 | ti/ab leisure-time OR free-time OR extracurricular | 24583 | |
| #5 | #1 AND #2 AND #3 AND 4 | 238 | |
| #6 | Add filter: Adolescent (keyword) | 130 | |
