# Supplementary material for: Increasing physical activity in sedentary adolescents through school-based interventions: a scoping review
Source: Front Sports Act Living. 2026 Jun 10;8:1736134. doi: 10.3389/fspor.2026.1736134 (PMC13291142; doi:10.3389/fspor.2026.1736134)
Supplement: Supplementary file 2 [file Supplementaryfile3.docx]

**Supplement 3. Outcomes categorized by intervention context**

| **Study ID** | **Outcome measure** | **Value** | **Change** |
| --- | --- | --- | --- |
| **Increased Physical Activity through Physical Education interventions (n = 14)** | | | |
| #3 | Vigorous activity leisure-time | γ010=0.53, SE=0.16, p=.003 | ↗ |
| #3 | Moderate activity | γ = 0.06, SE = 0.14, p = .67 | – |
| #4 | Doing any vigorous activity | OR = 6.97, CI [1.76, 27.58] | ↑ |
| #4 | Light activity | *d* = −0.80 | ↓ |
| #4 | Moderate activity | *d* = 0.65 | ↗ |
| #4 | Number of lifestyle activities | *d* = 0.76 | ↗ |
| #5 | Number of minutes completed 20SRT | *d* = 0.839 | ↑ |
| #6 | Leisure-time physical activity (LTPA) | g = 0.90 | ↑ |
| #7 | Moderate activity intervention | *d* = 1.23 | ↑ |
| #7 | Moderate activity time | ω² = .15 | ↑ |
| #7 | Vigorous activity time | ω² = .14 | ↑ |
| #7 | Vigorous activity intervention | *d* = 0.03 | – |
| #9 | Doing any vigorous activity | *d* = 0.72 | ↑ |
| #9 | Moderate activity | *d* = 0.38 | ↗ |
| **Increased Physical Activity through Community-based Interventions (n = 12)** | | | |
| #1 | Popular games | *d* = 0.35 | ↗ |
| #1 | Weekly time in MVPA | *d* = 0.17 | ↗ |
| #1 | Number of physical activities per week | *d* = 0.27 | ↗ |
| #2 | Moderate activity | β = −49.24 (SE = 22.18) | ↑ |
| #2 | Walking as leisure | β = −29.394 (SE = 14.61) | ↗ |
| #2 | Vigorous activity leisure-time | β = −64.09 (SE = 23.65) | ↗ |
| #2 | Active transportation | β = −36.34 (SE = 55.55) | – |
| #8 | LTPA younger boys | *d* = 0.28 | ↗ |
| #8 | LTPA older boys | *d* = 0.16 | – |
| #8 | LTPA younger girls | *d* = 0.02 | – |
| #8 | LTPA older girls | *d* = −0.02 | – |
| #10 | Effect of school type | *d* = −0.23 | ↓ |
| **Motivational Aspects (n = 3)** | | | |
| #5 | Sense of coherence | *d* = 0.630 | ↗ |
| #6 | Intention to be physically active | *g* = 0.82 | ↑ |
| #6 | Intention to be physically active girls | *g* = 0.64 | ↗ |
| Change legend: ↑ large increase; ↗ small or moderate increase; ↓ decrease; – no significant change.  Benchmarks were applied for Cohen’s d (0.2/0.5/0.8) (Cohen, 1988), odds ratios (1.22/1.86/3.0) (Chen et al., 2010), partial eta squared, and omega squared (0.01/0.06/0.14) (Cohen, 1988; Kirk, 1996). For regression coefficients (β, γ), reported magnitudes were interpreted descriptively in line with established guidelines (Gelman & Hill, 2007). | | | |
|  |  |  |  |
